# Management of bilateral pterygoid myositis ossificans-like lesion in dogs

**DOI:** 10.3389/fvets.2022.992728

**Published:** 2022-10-10

**Authors:** Mercedes De Paolo, Margherita Gracis, Giuseppe Lacava, Natalia Vapniarsky, Boaz Arzi

**Affiliations:** ^1^Dentistry and Oral Surgery Service, William R. Pritchard Veterinary Medical Teaching Hospital, School of Veterinary Medicine, University of California-Davis, Davis, CA, United States; ^2^Istituto Veterinario di Novara, AniCura, Novara, Italy; ^3^Department of Pathology, Microbiology and Immunology, School of Veterinary Medicine, University of California, Davis, Davis, CA, United States; ^4^Department of Surgical and Radiological Sciences, School of Veterinary Medicine, University of California, Davis, Davis, CA, United States

**Keywords:** pterygoid muscle, pseudoankylosis, ankylosis, mandibulectomy, computed tomography

## Abstract

Myositis ossificans (MO) and myositis ossificans-like lesions have been rarely described within the veterinary literature, and are even less common in the maxillofacial region. When MO affects the muscles of mastication, it can result in complete or partial inability to open the mouth. As with other conditions resulting in decreased or restricted mandibular range of motion, severe and potentially fatal sequelae such as difficulty with prehension, swallowing, and air exchange are possible. Diagnostic imaging is essential in achieving an accurate diagnosis and in formulating an appropriate treatment plan. In this “method” manuscript, we provide a detailed description of our approach to diagnosis and surgical management of MO-like lesions of the pterygoid muscles and describe our experience with two young French bulldogs.

## Introduction

Myositis ossificans (MO) is a term used to describe heterotopic bone formation within a muscle. In people, the disease is rare, and even more so in the maxillofacial region. The human medical literature has classically described MO as being either myositis ossificans progressiva (MOP; also known as fibrodysplasia ossificans progressiva) or myositis ossificans traumatica (MOT; also known as myositis ossificans circumscripta). The two are believed to have distinct etiologies and outcomes with MOP having a genetic underpinning and invariably progressing, whereas the mechanism of MOT is unknown but often suspected to occur secondary to trauma. In both processes, attempted excision of the heterotopic bone is often followed by recurrence (1–4).

Myositis ossificans is very rarely reported in dogs, and mostly in the limb muscles (5, 6). One report of suspected maxillofacial myositis ossificans in a dog was published (7), although it is unclear whether it truly reports MO or simply a pseudoankylosis subsequent to zygomatic arch fracture. Another more recent report describes a nearly identical lesion to those in our report, although it is not referred to as myositis ossificans (8). There are also several reports in cats of myositis ossificans progressiva (or fibrodysplasia ossificans progressiva), with one describing it in the maxillofacial region (9).

As with other conditions resulting in decreased mandibular range of motion, severe and potentially fatal sequelae are possible. Difficulty with prehension, swallowing, and air exchange are known life-threatening complications, in addition to decreased quality of life secondary to an inability to groom and play. For this reason, surgical intervention is imperative if euthanasia is to be avoided. However, based on the human medical literature, attempted excision of these osseous proliferations typically recur. In addition, excision of bridging osseous lesions from the base of the skull to the mandible carries the risk of catastrophic hemorrhage. Therefore, an alternative surgical technique which provides a functional outcome without the risk of recurrence or significant hemorrhage is needed.

In this report, the diagnostic imaging, clinical, and histopathologic features of two cases with MO-like lesions of the pterygoid muscles are described. In addition, a surgical approach of bilateral segmental mandibulectomies resulting in functional long-term outcome will be detailed and discussed.

## Diagnostic imaging techniques

**Figure 1:**
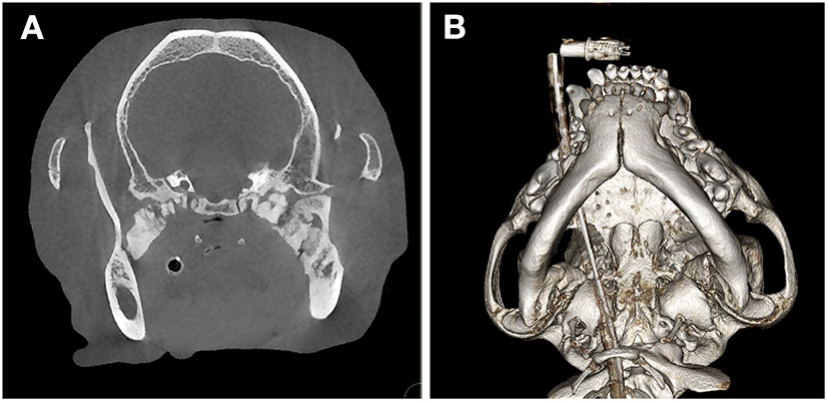
Cone beam CT images for the dog described in Case 1. **(A)** CT image demonstrating bilateral smoothly margined, dense osseous proliferation in the location of the lateral pterygoid muscles, spanning the region of the medial pole of the condylar process and tympanic bulla to the medial aspect of the caudal body of the mandible. **(B)** A 3D reconstruction of the same scan, viewed from the ventral aspect.

Two dogs presented for evaluation of a progressive inability to open the mouth at a young age and that did not exhibit severe atrophy, swelling, or pain associated with the muscles of mastication, suggestive of masticatory myositis, received a cone beam CT (NewTom 5G CBCT Scanner, NewTom, Verona, Italy) or CT (GE Optima 660, Milan, Italy) scan. Each case was evaluated on medical grade monitors. Findings of the CT/CBCT include bilateral smoothly margined, dense osseous proliferation spanning the region of the medial pole of the condylar process and tympanic bulla to the medial aspect of the body of the mandible (Figure 1).

## Surgical and anesthetic technique

### Anesthetic considerations

Depending on the severity of the dog's inability to open the mouth, modified anesthetic approaches are taken. In dogs with minimal or no opening of the mouth, a temporary tracheostomy is anticipated. Alternatively, in dogs with a small amount of remaining range of motion, flexible or rigid endoscopy is utilized to assist with orotracheal intubation. In all cases, preparations are made for a temporary tracheostomy if the endoscopy-assisted intubation is unsuccessful. Patients are pre-operatively blood-typed and cross-matched in the event of significant hemorrhage. Inferior alveolar nerve blocks may not be possible if there is obliteration of the mandibular foramina by proliferative osseous tissue. Post-operatively, patients are monitored in the intensive care unit for at least 12 h.

### Extraoral approach to segmental mandibulectomy

**Figure 2:**
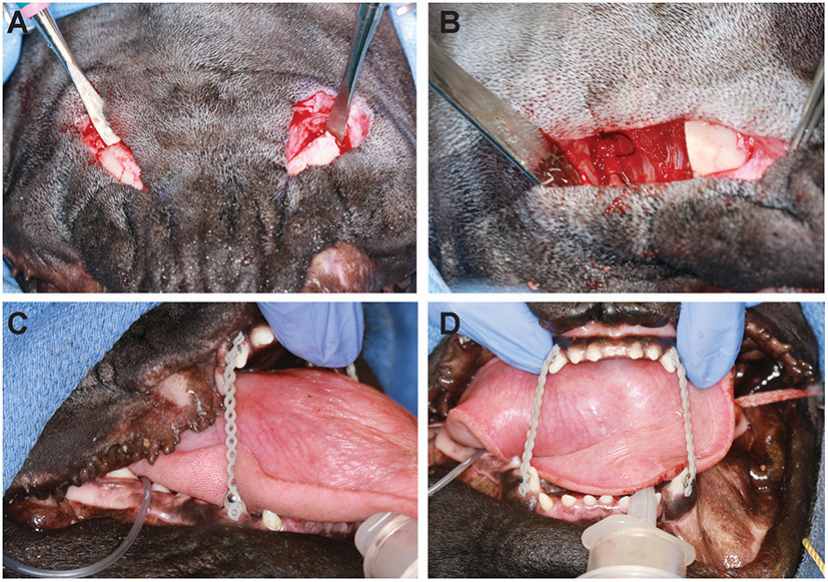
Intraoperative images of the extraoral approach to bilateral segmental mandibulectomy and placement of the elastic orthodontic training device in case 1. **(A)** Two skin incisions are made on the ventral aspects of the mandibles. **(B)** Osteotomies are created using a piezoelectric surgical unit, and the segment of bone is removed through the extraoral incision. **(C,D)** Orthodontic buttons are placed on maxillary and mandibular canine teeth, and elastic power chains are placed between them to help minimize ventral deviation during soft tissue healing.

Dogs are clipped along the entirety of the ventral and lateral aspect of both mandibles and aseptically prepared for surgery. The dogs are placed in dorsal recumbency with the neck gently extended. A skin incision is made using a #15 blade along the ventromedial border of each mandible, extending from the caudal aspect of the mandibular symphysis to just rostral to the angular process (Figure 2A). Then the subcutaneous fascia and platysma muscle are incised. A portion of the rostral belly of the digastricus muscle on the ventromedial mandible is elevated, but the insertions of the masseter muscle are left in place. Portions of the insertions of the mylohyoideus and genioglossus muscles are also elevated subperiosteally on the medial aspect of the mandible to facilitate exposure of the intended osteotomy sites. Alveolar mucosa and gingiva are elevated via the extraoral approach on the lingual and buccal aspects of the segment to be removed. If necessary, the sublingual branch of the facial artery and vein are ligated and transected.

Two hypodermic needles are inserted from the oral cavity, one along the buccal aspect of the tooth root intended to serve as the mesial osteotomy site and another along the buccal aspect of the tooth root intended to serve as the distal osteotomy site. Removing a critical-sized defect in order to prevent healing of the cut ends of the bone as far caudally as possible maximizes subsequent mouth opening. Using a piezoelectric surgery unit with an osteotomy insert and continuous irrigation (Acteon, Norwich, England; or Piezosurgery Touch^®^, Mectron, Carasco, Genova, Italy), shallow outlines of the bone cuts are made. The needles are then withdrawn and removed from the surgical site. The caudal osteotomies are made sequentially in both mandibles in case there is a need to open the mouth emergently. Osteotomies are then performed rostrally as well. Once both osteotomies are complete for each segment (rostral and caudal, right and left) and the inferior alveolar neurovascular bundles are ligated rostrally and caudally, the vessels and nerves are transected. Bone holding forceps are then used to place traction on the segments so that a #15 scalpel blade can be used to remove any remaining soft tissue attachments, including the oral mucosa and gingival attachment surrounding each mandibular first and/or second molar teeth. The segments are then removed through the extraoral incision (Figure 2B). Dental radiographs of the excised portion are obtained to ensure complete removal of the tooth and, if needed, extraction of remaining root fragments are performed prior to intraoral closure. Where indicated, osteoplasty is performed using a piezoelectric unit to remove sharp edges. The surgical sites are lavaged copiously with sterile saline or Ringer's lactate prior to closure. Subcutaneous tissues are closed with 4-0 poliglecaprone 25 in a simple interrupted pattern, and the skin is closed routinely.

### Intraoral closure and placement of elastic training device

After closing the extraoral incision, the dog's mouth is opened, and an intraoral examination is performed. The oropharynx is suctioned and a pharyngeal pack is placed. Intraoral dental radiography of the mandibles is performed to ensure no iatrogenic damage to surrounding teeth has occurred. The surgical sites are again flushed using sterile saline or Ringer's lactate prior to suturing the gingival tissues with 4-0 poliglecaprone 25 in a simple interrupted pattern.

An elastic training device is placed to provide support to the rostral portions of the mandible during soft tissue healing and fibrous tissue formation in the critical size defect created. After cleaning and etching each of the four canine teeth, orthodontic buttons are cemented on the buccal aspect of each tooth. Equal lengths of elastic orthodontic power chain are then placed between the mandibular and maxillary canine teeth on each side (Figures 2C,D). The length of chain between upper and lower canine teeth is subjectively assessed to be adequate when there is no ventral deviation of the rostral mandibles at rest and the tongue can freely be moved in and out. The power chains are changed weekly and maintained for at least 1 month.

## Histology

**Figure 3:**
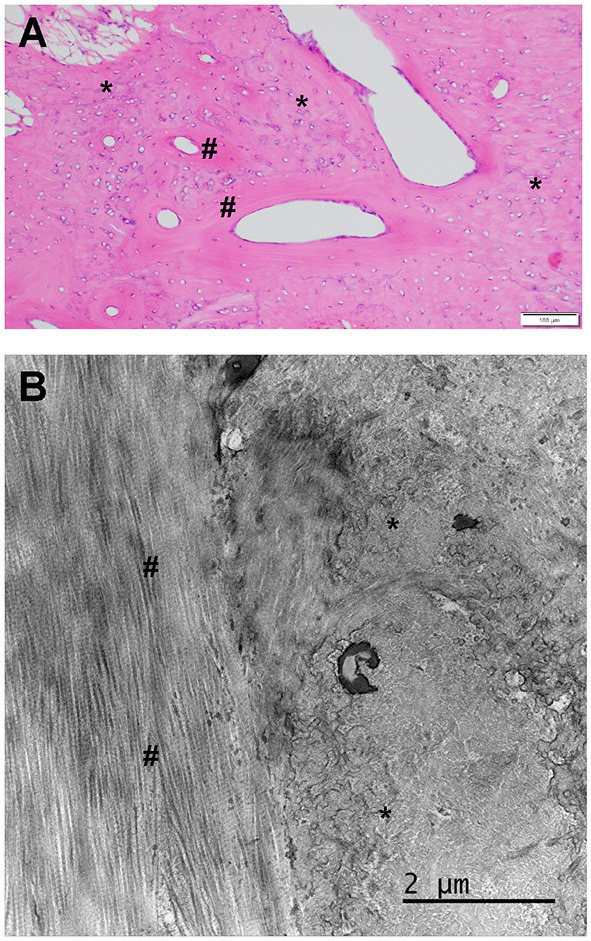
**(A)** Standard HE histology of the muscle biopsy from case 2. There is no apparent muscular tissue in the section presented. Instead, there is a mixture of mature lamellar (number sign) and immature woven bone (asterisk). The clear oval spaces were probably occupied by blood vessels that were lost in the processing. **(B)** Transmission electron microscopy for the interface between the woven (right, asterisk) and lamellar bone (left, number sign). Note the regimented and parallel collagen fiber alignment in the lamellar bone and the more haphazard and scrambled appearance of the collagen fibers in the woven bone. The dark areas represent mineralized portions of the bone matrix that were not completely removed by the decalcification process.

A biopsy was obtained from the right side using bone rongeurs in Case 2 for histologic analysis (Figure 3). The histomorphology of the submitted biopsied tissue was consistent with bone, and there was no evidence of muscle tissue in the examined sections. The osseous component is composed of lamellar (mature) and woven bone (immature) bone (Figure 3). The lamellar bone is characterized by regimented collagen fibers aligned in a circumferential fashion around a clear oval space. The fibers in these circumferential structures are aligned parallel and contain very few cells (osteocytes). The woven bone component comprises haphazardly arranged collagen matrix fibers and contains more numerous and more plump cells (osteoblasts). On the ultrastructural level (transmission EM), the fiber alignment patterns described above are clearly represented. In concert, these histologic features indicate heterotopic ossification or osseous metaplasia.

## Case reports

### Case 1

A 1-year-old male, intact French bulldog weighing 10.3 kg was presented for evaluation of progressive inability to open the mouth. The dog was found as a stray at ~4 months of age and was unable to completely open or close the mouth which progressed over the next 6 months. This resulted in severe difficulty prehending food as well as multiple episodes of aspiration pneumonia for which the dog required hospitalization. The owner reported frequent ptyalism, retching or gagging, and overt vomiting during and after eating. On presentation, the dog was bright, alert, and responsive with a body condition score of 3/9. Vital parameters, including body temperature, were within normal limits. Aside from stenotic nares and severe intermittent stertor accompanied by intermittently increased respiratory effort, general physical examination was unremarkable. Awake extraoral examination revealed a maximum interincisal distance of approximately 10 mm with apparent inability to close the mouth. The tongue protruded from the right side of the mouth. Muscles of mastication were symmetrical without evidence of swelling or atrophy. No pain could be elicited on palpation of maxillofacial structures or on attempted opening of the mouth. The dog had severe relative mandibular brachygnathism despite an overall brachycephalic skull conformation. Intraoral examination was limited due to inability to open the mouth, but the right mandibular dentition was noted to be buccoverted, presumably secondary to tongue protrusion. In addition, there was significant crowding and rotation of multiple teeth.

Based on physical exam and history, ankylosis or pseudoankylosis of the TMJ were suspected, and advanced imaging utilizing cone beam CT (CBCT) scan was elected. Given concerns for aspiration pneumonia and likely brachycephalic obstructive airway syndrome (BOAS), a sedated CBCT was ruled out. The dog was premedicated with maropitant (1 mg/kg SQ) the evening prior to anesthesia. Prior to anesthesia induction, it was sedated with butorphanol IM (0.3 mg/kg) and dexmedetomidime (4.85 mcg/kg IM). After an IV catheter was placed, the patient was induced with ketamine (4 mg/kg IV) and midazolam (0.2 mg/kg IV). Using a flexible endoscope for guidance, the dog was orotracheally intubated, although preparations for temporary tracheostomy were made prior to induction. Complete blood count and serum biochemistry did not reveal significant abnormalities.

Anesthetized interincisal distance was noted to be 10 mm, consistent with the awake exam. CBCT revealed bilateral bridging osseous proliferation spanning from the region surrounding the tympanic bulla to the medial aspect of the mandible in the region of the mandibular foramen (Figure 1). The osseous lesion contacted the medial pole of condylar processes bilaterally. Both temporomandibular joints were malformed with hypoplastic condylar processes, flattened mandibular fossae, and narrowed joint spaces. The tympanic bullae were hypoplastic, thickened, and filled with soft-tissue attenuating material bilaterally. The patient was noted to have an elongated and thickened soft palate.

A diagnosis of bilateral pseudoankylosis was made, suspected to be due to myositis ossificans-like lesions of the pterygoid muscles. A replica of the skull was 3D printed for surgical planning. After conversations with the owner regarding risks and benefits of various treatment options including excision of the osseous proliferation, medical management, and salvage bilateral segmental mandibulectomy, the latter option was elected. The dog was anesthetized using the protocol reported earlier with the addition of an intraoperative continuous rate infusion of sufentanil (0.01 mcg/kg/min). No locoregional anesthesia was performed. Ampicillin was administered once at the start of the procedure (20 mg/kg IV). Bilateral segmental mandibulectomy was performed as described above using an extraoral approach followed by intraoral closure and placement of elastic training (Figure 2). The removed segments measured 25 mm on each side. The dog recovered uneventfully aside from moderate dysphoria and several episodes of continued regurgitation. The sufentanil CRI was continued in the immediate post-operative period in addition to a dexmedetomidine CRI (0.5 mcg/kg/h) to aid in pain and anxiety control, but both were discontinued overnight. A fentanyl patch (25 mcg/h) had been placed at the conclusion of the procedure. The dog was hospitalized and monitored in the ICU for 18 h, during which time he remained stable and comfortable. He was bright, alert, and eager to eat the following morning and was subsequently monitored for an additional 24 h. During hospitalization, the dog was maintained on IV lactated Ringer's solution at a rate of 2 ml/kg/h until it ate independently, at which point IV fluids were discontinued. The dog was also maintained on the following IV medications for the first 36 h: carprofen (2.2 mg/kg q12 h), ampicillin (20 mg/kg IV q8 h), maropitant (1 mg/kg q24 h), and pantoprazole (1 mg/kg IV q12 h). Once the dog was eating independently and consistently without vomiting and/or regurgitation, transition to oral medications ensued. The dog was discharged with the following medications: amoxicillin/clavulanic acid (187.5 mg PO q12 h for 14 days), carprofen (37.5 mg PO q24 h for 10 days), and tramadol (50 mg PO q8–12 h as needed for pain starting 48–72 h after discharge). Instructions to hand feed small meatballs of wet food slowly for the first several weeks and to restrict activity as much as possible were sent home. In addition, the client was instructed to return with the dog to change the orthodontic chains in 1 week and again for suture removal and chain replacement in 2 weeks.

The elastic chains were maintained for ~4–5 weeks after surgery. The client reported moderate difficulty in changing and maintaining the chains but found that it was possible to replace them when the dog was sleeping. The dog's ability to eat improved rapidly, and the client reported less stertor, more energy, and a more playful demeanor after recovering from surgery.

2 months after the initial procedure, the dog returned for a periodontal treatment. An oral exam revealed mild dehiscence in both sites, but granulation tissue filled the mandibulectomy defects, with no bone exposure. The right mandibular fourth premolar tooth had significant gingival recession along the distal aspect. Buccoversion of all right mandibular dentition was still present. The buttons were removed and a periodontal treatment was performed. The right mandibular fourth premolar tooth was extracted, the granulation tissue was debrided, and the oral mucosa over the mandibulectomy area was closed routinely. The dog recovered well and continued to improve at home.

**Figure 4:**
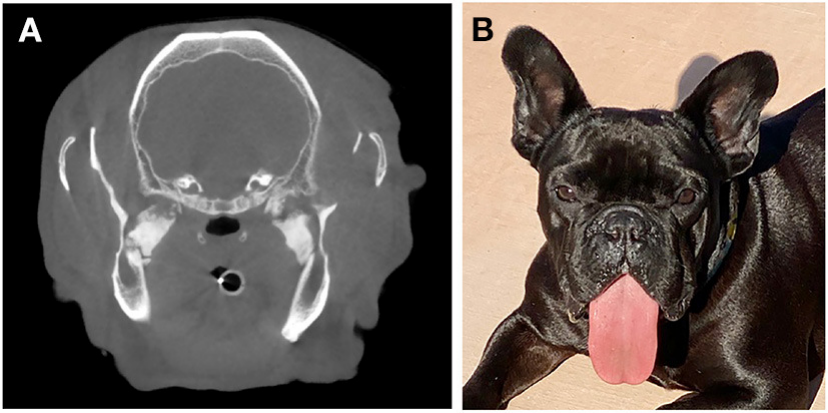
The dog described in Case 1 at its 3-year recheck exam. **(A)** Note the relatively static appearance of the lesion as demonstrated on the CT image as comparted to Figure 1. **(B)** Clinical image demonstrating the visual appearance 3 years following surgery.

3 years after the initial surgery (Figure 4), the dog returned for a CBCT and periodontal treatment. The dog was reported to be doing well and had regained the ability to pick up and bite down on objects within several months of the initial procedure. The tongue protruded and both rostral mandibles were ventrally deviated at rest. Anesthetized CBCT revealed static osseous proliferations relative to the original scan in the region of the pterygoid muscles and rounding of the margins of the mandibulectomy sites. At the time of publication, ~3.5 years after surgery, the dog was reported to be thriving at home with no ongoing concerns.

### Case 2

A 7-month-old, male intact, French bulldog weighing 8.2 kg was presented because of reduced ability to open the mouth. The dog had been adopted 5 months earlier and the new owner quickly noticed difficulties opening the mouth. A latero-lateral thoracic radiographic examination obtained a week before presentation showed normal cardiac and pulmonary structures. The dog was anesthetized to perform an endoscopic examination to stage his BOAS, which showed mild elongation of the soft palate, mild evertion of the laryngeal saccules, mild laryngeal collapse, and mild reflux esophagitis of the proximal esophagus. Reduced interincisal distance was identified during the procedure, and the dog was referred. At the time of presentation, the dog was reported to have a good appetite, to be able to eat and drink, but with frequent episodes of retching after drinking as well as seldom regurgitation and vomiting. The dog was also reported to have dyspnea, stridor, inspiratory efforts, exercise intolerance, and was reportedly unable to play with toys using his mouth but was bright, alert, and responsive with a body condition score of 5/9. A left sided grade I/VI heart murmur, mild stenotic nostrils and moderate stertor and stridor were present. An echographic examination diagnosed a mild mitral insufficiency that appeared hemodynamically insignificant.

A slight atrophy of the left muscles of mastication was present. Retropulsion of the eye globes was symmetrical and considered normal. No pain could be elicited on palpation of maxillofacial structures or on attempted opening of the mouth. Despite the brachycephalic conformation of the skull, a mild (8 mm) class 2 malocclusion was present, and the mandibles also appeared slightly rotated to the left side. Maximum interincisal distance appeared to be 10 mm. A permanent dentition with absence of left mandibular second and third molar teeth and right mandibular third molar tooth, and crowding and rotation of multiple teeth were evident. During examination, the dog regurgitated an abundant amount of clear fluid. Based on physical exam and history, TMJ ankylosis or pseudoankylosis were suspected.

A complete blood count and serum biochemistry were within normal limits, and 3 weeks later the dog was anesthetized to perform a CT scan with both pre- and post-contrast images available for evaluation. The ventral neck was clipped and prepared for a possible tracheostomy. After an IV catheter was placed, anesthesia was slowly induced with fentanyl (6 mcg/kg), propofol (3 mg/kg), and midazolam (0.2 mg/kg) IV, without premedication. Although a rigid endoscope was available to facilitate the procedure, endotracheal intubation was first attempted and performed blindly with a #3 cuffed endotracheal tube, which was considered slightly undersized for the patient but still effective in obliterating the tracheal lumen. Anesthesia was maintained with oxygen and isofluorane.

**Figure 5:**
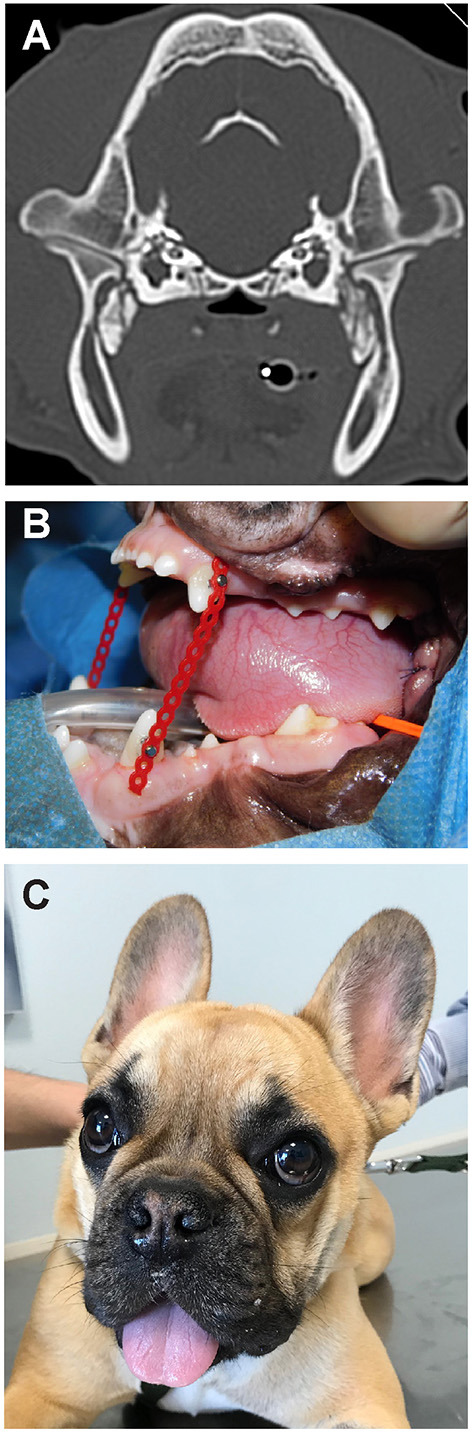
The dog from Case 2. **(A)** CT scan demonstrating bridging osseous proliferation at the area of the lateral pterygoid muscles as noted in case 1. **(B)** Placement of an elastic training device at the conclusion of surgery. **(C)** The same dog at a 3 week recheck exam following surgery.

Interincisal distance measured under anesthesia was confirmed to be 10 mm. The CT examination showed symmetrical, multilobular, tubular mineralizations of the right and left lateral pterygoid muscles, originating dorsally between the lateral wall of the tympanic bullae and the mandibular condylar process, and extending to the medial surface of the mandibular ramuses, just caudo-ventrally to the mandibular foramina (Figure 5A), which appered free from obstruction. The temporal and masseter muscles were considered slightly hypotrophic, more so on the left side. Also, mandibular brachygnathism, and bilateral TMJ dysplasia with agenesis of the retroarticular process of the temporal bones and flattening and deformation of the TMJ articular surfaces were present. Furthermore, signs of otitis media (i.e., soft-tissue attenuating material present in both bullae), agenesis of the left frontal sinus, and ectopic nasal turbinates in the choanae were noted. Finally, subchondral defects of the left condylar process were present. A diagnosis of bilateral pseudoankylosis secondary to myositis ossificans-like lesions of the lateral pterygoid muscles was then made. The dog recovered uneventfully from anesthesia and was discharged the same day.

A bilateral segmental mandibulectomy was performed a week later. The dog was anesthetized and intubated using the protocol reported earlier with the addition of an intraoperative administration of maropitant (1 mg/kg), and a continuous rate infusion of fentanyl (10 mcg/kg/h) and propofol (0.2 mg/kg/min). A lidocaine bolus (1 mg/kg IV) was administerd 10 min after induction. No locoregional anesthesia was performed. Cefazolin was administered once at the start of the procedure (20 mg/kg IV).

Bilateral segmental mandibulectomy was performed as described above, making the ostectomy cuts through the distal root of right and left mandibular first molar teeth, and distal to the mandibular second molar teeth. This resulted in a removal of segments which were 10 mm on each side. Because of slight sublingual swelling, dexamethasone was administered intraoperatively (0.25 mg/kg IV). Once the mouth could be opened, the endotracheal tube was removed and replaced with a larger (#3.5) cuffed endotracheal tube. The right and left first molar teeth were extracted and a post-operative intraoral radiographic examination of the surgical area was performed. However, because of anatomical conformation and muscle mineralization, placement of the radiographic plates lateral to the caudal mandibular segments was limited. The surgical sites were then sutured routinely. A postoperative CT scan was performed. Postoperative interincisal distance was 77 mm, and the elastic chains were positioned to allow a 30 mm mouth opening (Figure 5B).

The dog recovered uneventfully, and was monitored in the intensive care unit for the following 24 h. It was discharged the following day, with a prescription for cephalexin (120 mg PO q12 h for 5 days), meloxicam (0.8 mg PO q24 h for 7 days), tramadol (25 mg PO q8 h for 7 days), and 0.1% chlorhexidine gel orally 2–3 times daily for 4 weeks. A few extra elastic chains were provided to the owners, with the instruction to change them about once weekly or sooner if necessary. It was recommended to offer small meatballs or dry food slightly moistened with water, and to allow eating and drinking from an elevated bowl and/or from the faucet. Furthermore, the owner was instructed to restrict the dog's physical activity as much as possible, but to favor chewing activity and perform manual physiotherapy gently opening and closing the dog's mouth several times a day, to decrease the chances for indirect bone healing in the ostectomy site.

At the 3-weeks (Figure 5C) and 9-weeks re-examinations the dog was reported to be active, playful, able to eat softened dry food and drink water from an elevated surface or from the faucet without problems, and to show only mild drooling. The owner had been able to change the elastic chains as instructed without problems. On presentation the surgical wounds appeared completely healed, and the dental brackets and elastic chains were in place. Both rostral mandibles were ventrally deviated at rest and mild to moderate central lingual protrusion was evident. It was impossible to manually close the mouth, as if the segmental gap created surgically had healed with rigid or semi-rigid tissues, with the rostral portion of the mandible slightly angled ventrally.

The elastic chains were kept in place for about 7 months. Successive follow-ups were performed by telephone, until the 8-months re-check. During this time the diet was modified to dry kibble, without any significant problems. The dog was reported to be active and able to play with corks and rubber balls using his mouth. Open mouth and mild central lingual protrusion were still present. Maximum interincisal distance was measured to be 44 mm in an awake state, and complete manual closure of the mouth was still impossible. It was then recommended a further re-check in about 10 months, to perform a CT re-examination to evaluate the healing of the surgical sites and any progression of the muscular lesions. At the time of publication, 15 months after surgery, the dog was reported to be thriving at home with no ongoing concerns.

## Discussion

To the authors' knowledge, this is the first report on the surgical management of bilateral pterygoid myositis ossificans-like lesions in dogs. This report demonstrates several clinically relevant aspects in cases of MO-like lesions with subsequent treatment with bilateral segmental mandibulectomy. First, diagnostic imaging features of the MO-like lesions are largely consistent with reports of myositis ossificans in people. Second, salvage treatment by means of bilateral segmental mandibulectomy resulted in an acceptable long-term outcome as demonstrated in both case examples. Third, both dogs were young French bulldogs with unknown early history, indicating that both genetic and traumatic etiologies may be considered. Fourth, diagnostic imaging by means of CT or CBCT was essential for diagnosis, surgical planning, and follow-up. 3D printing is also a very useful addition in surgical planning. Finally, special considerations for perioperative and post-operative anesthetic monitoring and care are required for these patients.

In the cases presented here, the combination of clinical signs and diagnostic imaging, were consistent with reports of MO in humans. Histopathology confirmed that the biopsy obtained from the pterygoid muscle location in case 2 is consistent with bone tissue composed of woven (immature) and lamellar (mature) bone. The latter indicates a metaplastic process of bone formation, also known as heterotopic ossification. As has been found in humans, the predominant clinical sign in our patients was inability to open the mouth. Neither of the patients had swelling, overt pain, or were febrile as may be expected in other diseases processes that lead to inability to open the mouth. In people, swelling and pain are only found occasionally (2). A combination of imaging modalities, including CT, MRI, and doppler ultrasound, is frequently employed when diagnosing MO in people (4), and the CT/CBCT images described in this report were consistent with findings in people. The histopathological descriptions of pterygoid muscle ossification in humans are not very specific and are usually limited to a brief description indicating the presence of heterotopic foci of osteoid woven bone, occasional cartilage, and collagen fibers (10). Other reports documenting heterotopic muscular ossification in the appendicular skeleton state that histopathology varies based on the stage of evolution of the ossification process (11). Specifically a progression from mesenchymal metaplasia to immature bone and eventually mature bone is described (11). Thefore, the presence of lamellar and woven bone elements in the biopsy sample from case 2 in this report comprised some of the features described for MO in people. In our patients, there was no evidence of progressive heterotopic bone formation as would be expected in MO progressiva. In fact, for the first patient, a follow-up CBCT 3 years after the initial visit showed that the lesions were static. The lack of progression in the first patient indicates that the lesions are more likely to be consistent with MO circumscripta than MO progressiva (FOP). Alternatively, lack of progression may represent the end stage of the ossification process. In summary, many imaging, clinical and histologic features of pterygoid muscle pathology presented here bear a striking resemblance to MO in humans; however, documentation of the progressive evolution of this lesion from fibrous metaplasia to mature bone associated with muscle bundles would be necessary to confirm this diagnosis completely.

From a surgical standpoint, excision of the osseous proliferation would have been ideal because it would have, theoretically, allowed for a completely normal occlusion and return to function in the future. However, human data suggests that recurrence is likely, possibly because the surgery itself is a traumatic event and may incite further bone formation (1). An alternative treatment which has been described in the human medical literature as well as in the veterinary case described by Ellenberger and Snyder (8) is physical therapy. In general, physical therapy alone does not tend to result in a significantly increased range of motion in people, even with aggressive physical therapy regimens (2). Given how severely the interincisal distance was decreased in our patients, especially in combination with their brachycephalic conformations and persistent weight loss and aspiration pneumonia (Case 1), medical management was not considered a viable option. Therefore, bilateral segmental mandibulectomy was considered to be a possible “salvage” option, which would allow the tongue and air to move more freely without directly addressing the abnormal osseous proliferations. In fact, both patients recovered remarkably well and exibited a good quality of life despite their lack of normal masticatory muscle function. Surprisingly, one of the dogs has apparently regained enough muscle and jaw function to be able to close its mouth and pick up objects, while the other was able to pick up objects but is still unable to completely close the mouth. It may be that careful and judicious periosteal elevation of muscle insertions rostral to the ostectomy allowed for some continued function. It is important to note that in Case 2, where the size of the removed segments was smaller (10 mm) than those in Case 1 (25 mm), the inability to completely close the mouth is suspected to be due to a fibrous or osseous union of the cut ends. This highlights the need to create an adequately sized defect to prevent bone healing.

In humans, MO progressiva is known to have a genetic component, whereas MO traumatica/circumscripta is thought to have a traumatic basis. Interestingly, both of our patients were young French bulldogs, which raises the possibility of a genetic predisposition, although this may be a coincidence as the patient in a previous report (8) was an Airedale terrier. Given that Dog 1 had an unknown early history and was found as a stray, it is also possible that he experienced a traumatic injury which predisposed him to development of MO. The history of Dog 2 was not remarkable, but a traumatic event at an earlier age cannot be completely excluded. Further research in this area is needed for better understanding.

In both cases, advanced diagnostic imaging was essential for obtaining a presumptive diagnosis and formulating a treatment plan. As has been discussed elsewhere (12), there are multiple reasons that a dog may be unable to open or completely close the mouth including masticatory myositis, TMJ dysplasia or luxation, neoplasia, neurological abnormalities, and others. Although some differential diagnoses can be ruled out based on physical exam and history, the remaining differential diagnoses require advanced imaging. Additionally, in one of the cases, 3D printing allowed for refinement of the surgical plan and was helpful in educating the client on the surgical options and limitations.

Finally, the perianesthetic and perioperative periods required special consideration for both of our patients. The primary challenge in both cases was securing the airway, given that both had very limited interincisal distance. One required endoscope-guided orotracheal intubation, but in both cases emergency tracheotomy was anticipated. Once the surgery was completed, the mouth could be opened but given the brachycephalic conformation, the possibility of significant swelling leading to airway obstruction was still present. Therefore, careful monitoring in the ICU with or without oxygen supplementation for at least 12 h is recommended. In addition, intraoperative hemorrhage was a possibility. In general, when the inferior alveolar artery is inadvertently lacerated or transected prior to completion of the ostectomies, placing digital pressure over the mandibular foramen while ligating the artery typically helps control hemorrhage. However, in case 1, the mandibular foramen was occluded by bone, making digital compression impossible if needed. Therefore, it is advisable to ensure that appropriate blood products are easily available prior to undertaking this surgery. Additionally, the use of piezosurgical equipment, which is considered safer when working in proximity to important neurovascular structures (13), is even more advantageous in these cases.

## Conclusion

Myositis ossificans-like lesions of the muscles of mastication in dogs are rare but debilitating and potentially life-threatening disease processes. Given that these lesions have the same general and vague presenting complaint of ‘inability to open the mouth' as other disease processes, it is imperative that diagnostic imaging is obtained to achieve a presumptive diagnosis. Given the likelihood of recurrence following attempted surgical excision of MO-like lesions, an alternative surgical procedure, such as the one presented here, is needed. The complexity of these cases requires thorough planning in the perioperative period as it pertains to anesthesia, surgery, and post-operative care. However, this report demonstrates that salvage bilateral segmental mandibulectomy for patients with MO-like lesions of the pterygoid muscles may result in acceptable clinical outcome for patients with an otherwise life-threatening disease process.

## Data availability statement

The raw data supporting the conclusions of this article will be made available by the authors, without undue reservation.

## Ethics statement

Ethical review and approval was not required for the animal study because the study is retrospective in nature and included clinical cases, hence, it is exempt from IACUC requirements. The standard written informed consent was required for all procedures performed at the William R. Pritchard Veterinary Medical Teaching Hospital of the University of California, Davis was obtained from the owners. The standard written informed consent required for all procedures performed at both facilities.

## Author contributions

MD: surgical concept and design, manuscript writing, and provision of study cases. MG: manuscript writing, provision of study materials and cases, and review of the manuscript for important intellectual input. GL: manuscript writing and review of the manuscript for important intellectual input. NV: manuscript writing, interpretation of pathology and electron microscopy, and review of the manuscript for important intellectual input. BA: study concept, surgical concept and design, manuscript writing, provision of study materials and cases, and review of the manuscript for important intellectual input. All authors contributed to the article and approved the final version.

## Conflict of interest

The remaining authors declare that the research was conducted in the absence of any commercial or financial relationships that could be construed as a potential conflict of interest.

## Publisher's note

All claims expressed in this article are solely those of the authors and do not necessarily represent those of their affiliated organizations, or those of the publisher, the editors and the reviewers. Any product that may be evaluated in this article, or claim that may be made by its manufacturer, is not guaranteed or endorsed by the publisher.
